# Aqueous sage leave extract attenuates inflammation and oxidant-induced genotoxicity in human peripheral blood mononuclear cells

**DOI:** 10.2478/aiht-2024-75-3836

**Published:** 2024-06-29

**Authors:** Ana Valenta Šobot, Marijana Janić, Iva Popović, Tamara Lazarević-Pašti, Tatjana Momić, Aleksandar Krstić, Jelena Filipović Tričković

**Affiliations:** University of Belgrade, Vinča Institute of Nuclear Sciences, Department of Physical Chemistry, Belgrade, Serbia

**Keywords:** AChE inhibition, antigenotoxic effects, anti-inflammatory effects, antioxidant, *Salvia officinalis* L, antigenotoksičnost, inhibicija AChE, protuupalni učinci, antioksidans, kadulja, *Salvia officinalis* L.

## Abstract

Traditional medicine has used sage (*Salvia officinalis* L.) preparations for centuries to prevent and treat various inflammatory and oxidative stress-induced conditions. The aim of this *in vitro* study was to determine the bioactive properties of a sage leave extract obtained with environmentally friendly aqueous extraction and lyophilisation in primary human peripheral blood cells. To that end we measured the total phenolic and flavonoid content (TPC and TFC, respectively) with gas chromatography-mass spectrometry (GC-MS). Non-cytotoxic concentrations determined with the trypan blue assay were used to assess the antioxidant (DPPH, ABTS, and PAB assay), antigenotoxic (CBMN assay), immunomodulatory (IL-1β and TNF-α), and neuroprotective effects (AChE inhibition). The extract contained high TPC (162 mg GAE/g of dry extract) and TFC (39.47 mg QE/g of dry extract) concentrations, while β-thujone content was unexpectedly low (below 0.9 %). Strong radical-scavenging activity combined with glutathione reductase activation led to a decrease in basal and H_2_O_2_-induced oxidative stress and DNA damage. A decrease in TNF-α and increase in IL-1β levels suggest complex immunomodulatory response that could contribute to antioxidant and, together with mild AChE inhibition, neuroprotective effects. Overall, this study has demonstrated that aqueous sage leave extract reduces the levels of thujone, 1,8-cineole, pinene, and terpene ketones that could be toxic in high concentrations, while maintaining high concentrations of biologically active protective compounds which have a potential to prevent and/or treat inflammatory and oxidative stress-related conditions.

## Sažetak

*Salvia officinalis* L. stoljećima se koristi u tradicionalnoj medicini za prevenciju i liječenje raznih upalnih i oksidacijskim stresom izazvanih poremećaja. U ovoj studiji željeli smo ekstrahirati kaduljino lišće korištenjem ekološki prihvatljivog, “zelenog” pristupa vodenom ekstrakcijom i liofilizacijom te odrediti njegova bioaktivna svojstva u primarnim ljudskim perifernim krvnim stanicama. Ukupni sadržaj fenola i flavonoida i GC-MS korišteni su za karakterizaciju ekstrakta. Necitotoksične koncentracije, određene metodom bojenja s bojom tripan plavo, analizirane su za procjenu antioksidacijskih (DPPH, ABTS i PAB test), antigenotoksičnih (CBMN test), imunomodulacijskih (IL-1β i TNF-α) i neuroprotektivnih učinaka (AChE inhibicija). Ekstrakt je sadržavao visoku koncentraciju ukupnih fenola (162 mg GAE/g liofilizata) i flavonoida (39,47 mg QE/g liofilizata), dok je sadržaj β-tujona bio neočekivano nizak (niži od 0,9 %). Snažna aktivnost hvatanja radikala u kombinaciji s aktivacijom glutation reduktaze dovela je do smanjenja bazalnog i H_2_O_2_ induciranog oksidacijskog stresa i oštećenja DNA. Smanjenje TNF-α i povišenje razine IL-1β sugeriraju kompleksan imunomodulatorni odgovor koji bi mogao pridonijeti antioksidacijskim i, zajedno s blagom inhibicijom AChE, neuroprotektivnim učincima. Sveukupno, ova je studija pokazala da vodena ekstrakcija kaduljina lišća smanjuje toksične spojeve kao što su tujon, 1,8-cineol, pinen i terpenski ketoni, a održava visoku koncentraciju biološki aktivnih zaštitnih spojeva u ekstraktu, što bi moglo imati potencijal za prevenciju i/ili liječenje oksidacijskih i upalnih poremećaja.

Sage (*Salvia officinalis* L.), has long been used by traditional medicines worldwide both topically, to treat skin inflammation or sore throat, or systemically, in the form of an infusion, to treat inflammation and cognitive disorders (1, 2, 3, 4). These anti-inflammatory, antioxidant, antigenotoxic, neuroprotective, and many other pharmaceutical activities of sage have been confirmed by scientific research (3, 5, 6, 7) and are owed to bioactive compounds such as phenolic acids (rosmarinic, vanillic, ferulic, and caffeic acid), flavonoids (luteolin, apigenin, and quercetin), and monoterpenes (α- and β-thujone, 1,8-cineole, pinene, and camphor) (4).

The most common sage extract preparations are those in ethanol, methanol, or essential oil, given the high efficiency of the extraction process (3). However, these extracts can contain organic solvents or high monoterpene concentrations that can be toxic (8) and cause adverse effects as reported for thujone and camphor (9, 10), or 1,8-cineole and pinene (11), despite their antimutagenic or neuroprotective effects. *In vitro* and *in vivo* studies (12, 13) have demonstrated time and concentration-dependent monoterpene toxicity caused by high reactive oxygen species (ROS) formation, lipid peroxidation, and genotoxicity.

Considering the growing interest in the development of environmentally friendly extraction procedures to prepare extracts with good antioxidant, geno- and neuroprotective, and immunomodulatory effects that do not entail adverse toxicities, the aim of our study was to analyse the properties of a lyophilised sage leave extract obtained by extraction with deionised water and to test its biological effects on primary human peripheral blood cells. Our hypothesis was that aqueous extraction could provide high concentrations of biologically active protective compounds and reduce the levels of compounds that could be toxic in high concentrations.

To that end we used a range of non-cytotoxic sage extract concentrations to assess their antioxidant, antigenotoxic, immunomodulatory, and neuroprotective effects. To the best of our knowledge, this is the first such investigation in primary human blood cells.

## MATERIALS AND METHODS

### Aqueous sage extract preparation

Dried sage leaves (serial number: 24100920) purchased from the Dr Josif Pančić Institute of Medicinal Plant Research, Belgrade, Serbia, were ground, poured over with boiling deionised water (10:1 water-to-sage ratio), left for 90 min at room temperature, and filtered through a 5–13 μm pore filter paper (Lab Logistic Group, Meckenheim, Germany). The obtained extract was frozen at −20 °C and freeze-dried under a vacuum pressure of 400 Pa to obtain a dry lyophilisate. The lyophilisate was then dissolved in deionised sterile water and filtered through a 0.2 μm Minisart^®^ filter (Sartorius, Göttingen, Germany).

### Determination of total phenolic and flavonoid content

The total phenolic and flavonoid content (TPC and TFC, respectively) of the aqueous sage extracts was determined as described by Singleton et al. (14) and Chang et al. (15), respectively, with slight modifications adapted for 96-well microplates. Gallic acid (Sigma-Aldrich, St. Louis, MO, USA) was used to construct the calibration curve for TPC measurements, and its concentrations in water ranged from 0.625 μg/mL to 80 μg/mL, while the sage extract was analysed in concentrations of 0.125, 0.25, and 0.5 mg/mL. Quercetin (Sigma-Aldrich) was used to construct the calibration curve for TFC in concentrations ranging from 0.625 μg/mL to 80 μg/mL, while the sage extract was analysed in concentrations of 0.75, 1.25, and 1.75 mg/mL. The absorbance for TPC was read at 700 nm and for TFC at 415 nm on a microplate reader (Sunrise, Tecan Group Ltd, Männedorf, Switzerland). TPC is expressed as mg of gallic acid equivalents (GAE) per g of dried extract (de), and TFC as mg of quercetin equivalents (QE) per g of dried extract (de).

### Gas chromatography-mass spectrometry (GC-MS)

GC-MS was used to identify the most abundant compounds in the aqueous sage extract. To that end we dissolved the lyophilisate in 1 mL of deionised water, separated the organic aliquot by liquid-liquid extraction with n-hexane, and analysed it with an Agilent 7890B gas chromatograph coupled with a 5977 MSD mass detector and equipped with an HP-5 MS inert capillary column

[1] $$\mathrm{Inhibition}\;(\%)=\frac{A_{\text{control}}-A_{\text{sample}}}{A_{\text{control}}}\times 100$$

where A_control_ is the absorbance of control, and A_sample_ is the absorbance of the sample.

### Isolation and cultivation of human peripheral blood mononuclear cells (PBMC)

To assess the biological effects of aqueous sage extracts, we collected 10 mL of peripheral blood from each of the three healthy male volunteers aged 30–40 years in Li-heparin vacutainers (Becton Dickinson, Plymouth, UK). All volunteers read and signed the informed consent. The study was conducted in accordance with the Declaration of Helsinki and was approved by the Ethics Committee of the Vinča Institute of Nuclear Sciences, Serbia (approval No. 116-5-2/2023-000).

In whole blood we ran the cytokinesis block micronucleus (CBMN) assay, and measured interleukin levels and glutathione reductase (GR) activity. In PBMCs isolated from whole blood we evaluated cell viability and ran the prooxidant-antioxidant balance (PAB) assay.

PBMCs were isolated in a lymphocyte separation medium (Capricorn Scientific, Ebsdorfergrund, Germany) and resuspended in a RPMI 1640 medium (Capricorn Scientific) supplemented with 10 % foetal bovine serum (FBS, Capricorn Scientific) and 1 % penicillin-streptomycin (Gibco, Thermo Fisher Scientific, Waltham, MA, USA). The seeding density was 1×10^6^ viable cells/mL. All treatments with sage extracts in concentrations ranging from 0.075 to 2.25 mg/mL lasted 24 h.

### Trypan blue exclusion assay

After sage extract treatment we ran the trypan blue (TB) assay following the procedure described by Strober (19) to determine the range of non-cytotoxic concentrations. Equal volumes of PBMC suspension and 0.4 % trypan blue dye were mixed and applied to a haemocytometer (Cambridge Instruments Inc., Buffalo, NY, USA). Cell viability is expressed as a percentage of viable cells compared to untreated control (100 %).

### Quantitative assay for measuring glutathione reductase activity

Glutathione reductase (GR) is an intracellular enzyme that restores intracellular glutathione (GSH) by reducing glutathione disulphide (GSSG) in the presence of nicotinamide adenine dinucleotide phosphate (NADPH) and flavin adenine dinucleotide (FAD). GR activity (mU/mL) was measured in whole blood samples treated with aqueous sage extract as described by Mannervik (20) and calculated using the following Equation 2:

[2] $$\mathrm{GR}\;\mathrm{activity}\;(\mathrm{mU}/\mathrm{mL})=\frac{\Delta A\times V_{R}\times F}{V_{S}\times 6.22}\times 1000$$

where ΔA is the difference between control and sample absorbance; V_R_ the reaction mixture volume; F the dissolving factor; V_S_ substrate volume, and 6.22 the extinction coefficient.

### Prooxidant-antioxidant balance (PAB) assay

The effects of the aqueous sage extract on the oxidative status of PBMCs were assessed with the PAB assay as described by Alamdari et al. (21). The PBMCs were pre-treated with the extract 5 h prior to H_2_O_2_ treatment (200 μmol/L) to evaluate the ability of the extract to reduce the oxidative stress.

Optical density (OD) was measured at 450 nm (reference wavelength was 570 nm) on an absorbance microplate reader (Sunrise). PAB was expressed as percentage relative to control.

### Cytokinesis block micronucleus assay (CBMN)

The genotoxicity of the aqueous sage extract and its antigenotoxic potential against H_2_O_2_-induced damage were assessed with the CBMN assay as described by Fenech (22). Aliquots of whole blood were grown in the RPMI 1640 medium supplemented with phytohaemagglutinin-M (PHA-M, Capricorn Scientific) to stimulate lymphocyte proliferation.

Cell cultures were treated with a range of non-cytotoxic sage extract concentrations to assess genotoxicity and pre-treated with the same concentrations 5 h before the addition of 200 μmol/L H_2_O_2_ to assess the extract's genoprotective potential. Untreated cells served as a negative and H_2_O_2_-treated cells as positive control. Micronuclei were scored on at least 1,000 binuclear cells using the AxioImager A1 (Carl Zeiss, Jena, Germany) microscope with magnifications of 400× and 1000×, according to the scoring criteria set by the International Human Micronucleus (HUMN) Project (23).

The cytokinesis-block proliferation index (CBPI) was calculated using the following Equation 3:

[3] $$\mathrm{CBPI}=\frac{\left[\mathrm{MI}+2\mathrm{MII}+3(\mathrm{MIII}+\mathrm{MIV})\right]}{N}$$

where MI–MIV is the number of cells with 1–4 nuclei, and N the number of scored cells.

### Determination of pro-inflammatory cytokine levels

The effects of non-cytotoxic concentrations of sage extract on inflammation were determined by measuring the levels of interleukin 1β (IL-1β) and tumour necrosis factor α (TNF-α) in PHA-stimulated whole blood cell cultures. PHA is used to induce inflammation, as reported earlier (24, 25). IL-1β and TNF-α in cell culture supernatants were determined using the commercial LEGEND MAX™ Human IL-1β and TNF-α ELISA Kit (BioLegend, Inc., San Diego, CA, USA) according to the manufacturer's instructions. The OD values at 450 nm were measured using a microplate reader (Sunrise). The results are presented as pg/mL of IL-1β and TNF-α.

### Measurement of acetylcholinesterase (AChE) activity

To determine the effect of the sage extract on AChE activity we used a modified Ellman method as described elsewhere (26, 27). Free AChE from electric eel (2 U/mL) was incubated with sage extract in 50 mmol/L of phosphate buffer (pH 8) at 37 °C for 15 min. After that, we started the reaction by adding 75 mmol/L of acetylthiocholine iodide as enzyme substrate and 0.1 mmol/L of 5,5′-dithio-bis (2-nitrobenzoic acid) (DTNB) as chromogenic reagent. The reaction went for 5 min and was stopped with 10 mmol/L of sodium dodecyl sulphate (SDS). The formed product, 5-thio-2-nitrobenzoic acid (TNB), was measured spectrophotometrically (Lambda 35 UV-Vis Spectrometer, Perkin Elmer) at 412 nm.

### Statistical analysis

All experiments were performed in triplicate, and the results are presented as means ± standard deviations (mean±SD). We ran the analysis of variance ANOVA on the SPSS software for Windows 10, version 20 (IBM, Armonk, NY, USA). The level of significance is set to <0.05.

## RESULTS AND DISCUSSION

The total phenolic content of the sage extract investigated in this study was 162±3.51 mg GAE/g de, while the total flavonoid content was 39.47±4.31 mg QE/g de. Aqueous extraction yielded similar results as the methanol and ethanol extracts, which are considered the most efficient extraction agents (28, 29). To the best of our knowledge, there are but a few published papers describing water extraction of polyphenols and flavonoids from sage (30, 31, 32), and our extraction yield was higher.

GC-MS analysis identified 12 compounds, representing 98.06 % of the total sage leaf composition (Table 1). The main terpenoids in the extract were oxygenated monoterpenes camphor (15.47 %), α-terpineol (0.93 %), and β-thujone (0.89 %). The results for camphor content are within the published range from 5.08–25 % for different types of extracts (33, 34). The reported α- and β-thujone content, ranging from 7.07 to 21.85 % (35), is higher than in our aqueous extract. In fact, the content of β-thujone in our extract is very low. The literature data quite vary in this respect (33, 36, 37), as thujone content varies with drying conditions, harvesting time, seasonal variations, and geographic and climatic conditions. What may also influence sage extract composition are the temperature, time, solvent type, and particle size (38). Our extract was prepared by grinding and pouring it over with boiling deionised water, so we assume that the low thujone content and undetected 1,8-cineole and pinene are owed to grinding and high temperature, which increases evaporation of volatile components (3). In any case, given the reported toxicity (12, 13), low concentrations of monoterpenes found in our aqueous sage extract could be an advantage, as this lowers the risk of adverse effects.

**Table 1:** Relative percentage of identified compounds in the aqueous sage extract

**No.**	**Components**	**RRI**	**RT**	**Peak area (%)**
1	α-terpineol	1196	5.14	0.93
2	β-thujone	1103	5.93	0.89
3	Camphor	1143	6.36	15.47
4	endo-Borneol	1167	6.57	2.89
5	α-Bornyl acetate	1288	10.00	7.58
6	Acetophenone	1065	10.30	7.37
7	2,4 Di-tert butyl phenol	1519	11.13	7.05
8	Palmitic acid	1968	15.71	18.71
9	Linoleic acid	2172	17.10	8.16
10	Monopalmitin	2498	17.65	3.74
11	3-hydroxypropyl palmitate	2361	18.96	11.89
12	α-glycerol monostearate	2806	20.00	13.39

RRI – relative retention indices; RT – retention time

In turn, we determined high concentrations of palmitic (18.71 %) and linoleic acid (8.16 %), similar to other reported data (39).

**Figure 1:**
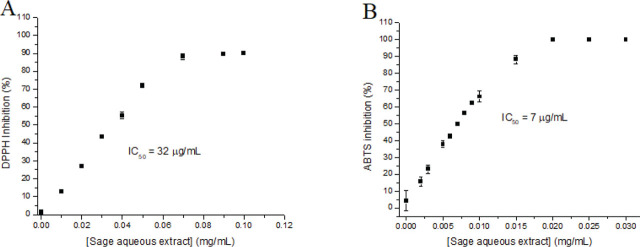
Scavenging properties of aqueous sage extract determined with the DPPH^•^ (A) and ABTS^•+^ assay (B) and expressed as a percentage of inhibition compared to control

Figure 1 shows that our aqueous sage extract inhibited the DPPH^•^ radical in a concentration-dependent manner, reaching the highest inhibition of 90 % with the concentration of 0.07 mg/mL. The calculated IC_50_ value was 0.032 mg/mL. The literature reports that the radical-scavenging activity of different sage extracts and essential oils varies greatly, from 116.5 μg/mL to 6.7 mg/mL, and that aqueous extract is the most potent (40, 41, 42). Our results are within the reported range but lower than those reported for the aqueous extract.

The IC_50_ value of ABTS^•+^ radical scavenging activity was 7 μg/mL. The maximum inhibition of 60 % was achieved with the concentration of 0.02 mg/mL (Figure 1B). IC_50_ values reported in other studies range from 50.79 μg/mL to 88.2±2.3 μg/mL (40, 41). Stronger ABTS^•+^ radical inhibition by our aqueous extract compared to other studies is in line with the DPPH^•^ scavenging results and reveals potent antioxidant activity, most likely owed to the high phenolic and flavonoid content (30, 31).

**Figure 2:**
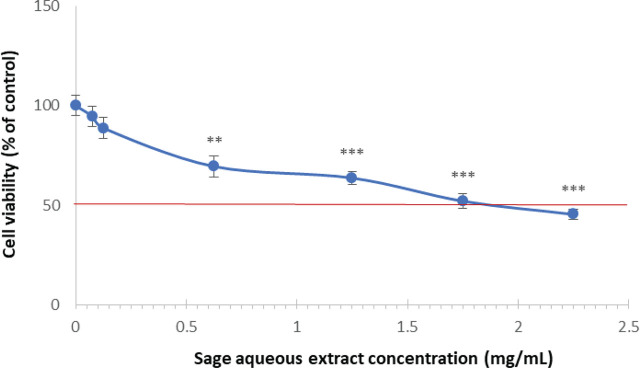
Viability of peripheral blood mononuclear cells treated with different concentrations (0.075–2.25 mg/mL) of aqueous sage extract for 24 h compared to untreated control. **P<0.01; ***P<0.001

Figure 2 shows concentration-dependent extract cytotoxicity measured with the TB assay, which was significant at 0.625 mg/mL (69.47 % of viable cells, *P*<0.01) and 1.25 mg/mL (63.67 % of viable cells, *P*<0.001). The IC_50_ value was achieved at 1.8 mg/mL, so only lower concentrations (0.075–1.25 mg/mL) were selected to test the genotoxic, antigenotoxic, antioxidant, and anti-inflammatory properties of our sage extract.

There are several reports of sage cytotoxicity as mostly concentration-dependent but also dependent on the extraction procedure and cell type used in the experiments. Vieira et al. (40) reported cell-type-specific cytotoxicity of ethanol and aqueous extracts, which was lower with the aqueous extract. Similarly, other studies (41, 43) reported concentration-dependent cytotoxicity of methanol extracts ranging from 50 μg/mL in malignant cell lines to more than 800 μg/mL in the control HUVEC cell line, or to 5.7 mg/mL for an ethanol extract in the HepG2 cell line. To the best of our knowledge, no study has yet reported the viability of PBMCs exposed to sage extracts. Lower IC_50_ values obtained in our study are probably owed to the physiological characteristics of PBMCs, as we speak of terminally differentiated cells that cannot progress through the cell cycle and divide without phytohaemagglutinin (PHA) stimulation. This is why we had to further evaluate the effects of non-cytotoxic aqueous sage extract concentrations on the cell proliferation of PHA-stimulated PBMCs with the CBMN assay (see below).

**Figure 3:**
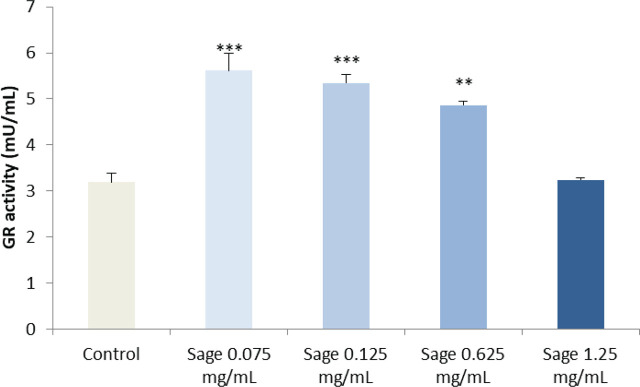
Glutathione reductase activity (mU/mL) in untreated control whole blood samples and those treated with increasing concentrations of aqueous sage extract. **P<0.01; ***P<0.001

The antioxidant potential of the sage extract was determined by measuring GR activity in the whole blood samples treated for 24 h. GR activity was significantly elevated at the concentrations ranging from 0.075 to 0.625 mg/mL (Figure 3). On the contrary, the highest tested concentration decreased GR activity, probably due to lower cell viability (Figure 2), limited cellular uptake capacity and/or the ability of sage extract components to activate the enzyme (41).

**Figure 4:**
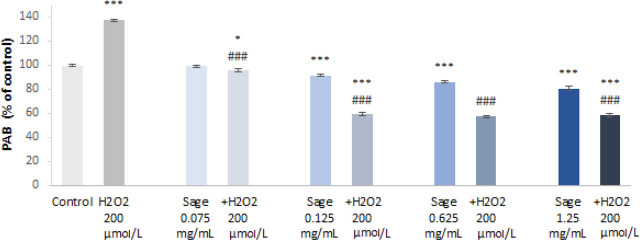
Prooxidant-antioxidant balance in untreated (control) peripheral blood mononuclear cells vs those treated with H_2_O_2_, aqueous sage extract, and their combination (presented as a percentage of untreated control). *P<0.05; ***P<0.001 – treatments vs. untreated control. ^###^*P*<0.001 – sage pre-treatments vs. H_2_O_2_ treatment

So far, we demonstrated that our sage extract had an antioxidant effect through a strong radical-scavenging activity and activation of GR, and yet it did not induce oxidative stress. In the experiments that followed we examined the effect of extract pre-treatment on H_2_O_2_-induced genomic damage and oxidative balance. As expected, PBMC treatment with 200 μmol/L of H_2_O_2_ led to a significant increase in PAB values compared to control (*P*<0.001) (Figure 4). However, pre-treatment with the sage extract lowered the PAB to control levels, and the protective effect increased with extract concentration. In fact, the extract lowered PAB values even in the cells not treated with H_2_O_2_, and this effect was significant starting with the concentration of 0.125 mg/mL. These results are in agreement with our DPPH^•^, ABTS^•+^, and GR activity findings and indicate that the aqueous sage extract can protect against ROS-induced oxidative damage.

**Figure 5:**
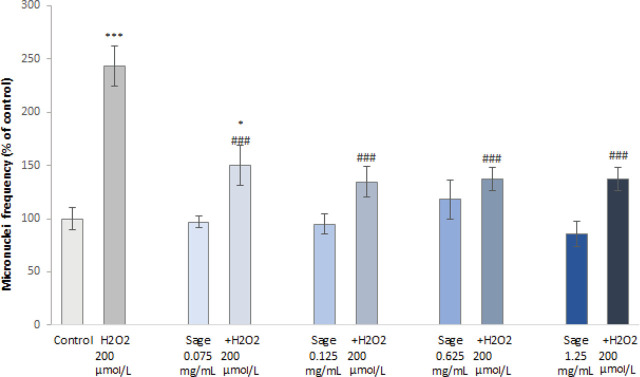
Micronucleus frequency in peripheral blood cells: untreated controls, H_2_O_2_-treated, sage extract-treated, and pre-treated with sage extract followed by H_2_O_2_ (presented as a percentage of untreated control). *P<0.05, ***P<0.001 – treated vs. control cells. ^###^*P*<0.001 – sage extract pre-treatment vs. H_2_O_2_ treatment

As for the genotoxic effects, the frequency of micronuclei (MN) in PHA-stimulated PBMCs treated with the sage extract did not differ significantly from untreated control (Figure 5). In the cells treated with H_2_O_2_ (200 μmol/L) it increased significantly, but pre-treatment with the sage extract countered this effect even at the lowest tested concentration, while the higher concentrations lowered MN frequency to near control values.

**Figure 6:**
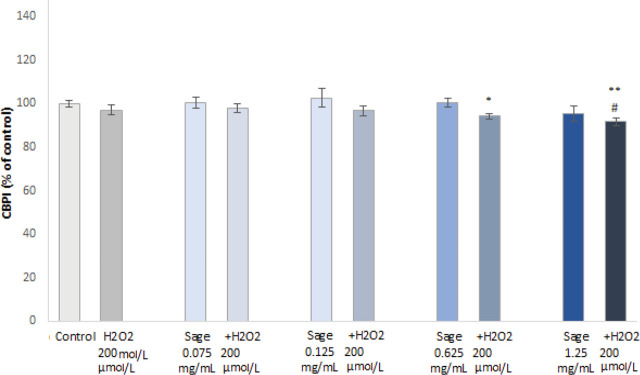
Cytokinesis-block proliferation indices (CBPI) of peripheral blood cells: untreated controls, H_2_O_2_-treated, sage extract-treated, and pre-treated with sage extract followed by H_2_O_2_ (presented as a percentage of untreated control). *P<0.05, ** *P*<0.01 – treated cells vs. control cells. ^#^*P*<0.05 – sage extract pre-treatment vs. H_2_O_2_ treatment

Figure 6 shows that the proliferation of PBMCs treated with H_2_O_2_ (200 μmol/L) or sage extract (regardless of the concentration) does not significantly differ from control. However, sage extract pre-treatment significantly lowered proliferation at the highest tested concentrations compared to control or H_2_O_2_ treatment. A possible explanation of this phenomenon might be in the pro-apoptotic activity of sage through the mitochondrial/caspase pathway (44, 45), which could only be achieved at higher extract concentrations.

**Figure 7:**
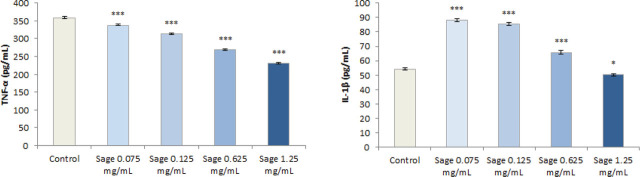
Pro-inflammatory cytokine levels, IL-1β (A) and TNF-α (B), in PHA-stimulated peripheral blood cells treated with aqueous sage extract in different concentrations. * *P* <0.05, ***P<0.001 – treated cells vs. control cells

Figure 7 shows the anti-inflammatory effects of the aqueous sage extract in PHA-stimulated peripheral blood cells. There is a concentration-dependent decrease in TNF-α levels, significant at all tested concentrations (*P*<0.001). Conversely, IL-1β levels increased, and the highest increase occurred at the lowest extract concentration (*P*<0.001). Only with the highest tested concentration did its levels drop to below control. Since both cytokines are involved in the same immune response pathway, these results are unexpected. Most research reports inhibition of IL-1β, IL-6, and TNF-α (46, 47, 48, 49). In our study, the opposite response of IL-1β and TNF-α to sage extract treatment may indicate the activation of other independent immunomodulatory pathways and polypharmacological effects owed to the extract's complex composition. Such response was also reported by Margetts et al. (11), who found that their ethanol sage extract decreased TNF-α but increased IL-6 levels. Perhaps this is owed to different cell types in full peripheral blood which are involved in cytokine production (50). Although a certain pro-inflammatory effect of the sage extract at the lower concentrations cannot be eliminated, opposite TNF-α and IL-1β levels suggest a complex immunomodulatory response that needs to be further elucidated.

Earlier studies reported the neuroprotective effects of sage through cholinergic binding properties *in vitro* (51, 52, 53). Our study has shown mild AChE inhibition, which started only with higher sage extract concentrations (Table 2). It was not possible to determine IC_50_ values under the given experimental conditions, since the highest tested dose of 10 mg/mL did not reduce 50 % of AChE activity. AChE inhibition by sage is mainly attributed to monoterpenes, including 1,8-cineole and α-pinene, which are abundant in its essential oils but have much lower concentrations in aqueous extracts (11). Our GC-MS analysis did not detect either of these monoterpenes, which is why AChE inhibition was low. However, the mild enzyme inhibition may also have been a result of high TPC, since phenolic acids, such as caffeic and rosmarinic acid, seem to inhibit AChE activity (54, 55). In any case, our study shows that aqueous extraction lowers the neurotoxicity of sage leaves and enhances their neuroprotective potential.

**Table 2:** AChE inhibition expressed as percentage of inhibited control activity

**Extract concentration (mg/mL)**	**AChE inhibition (%)**
0.075	0
0.125	0
0.625	16
1.25	31
10	41

## CONCLUSION

The advantages of aqueous extraction and lyophilisation of sage leaves are numerous. Aqueous extraction removes the environmental and cytotoxic risks pertinent to organic solvents used as extraction agents, yet yields high concentrations of beneficial bioactive compounds and lowers the concentration of those potentially toxic. High phenolic and flavonoid content provides strong anti-oxidative defence and genoprotection to human peripheral blood cells, which, combined with immunomodulatory properties, might contribute to the prevention of oxidative stress and inflammatory-related diseases. Additionally, AChE inhibition is mild, which points to the extract's neuroprotective role. However, caution is advised with doses used in medicinal preparations, as concentrations higher than 0.625 mg/mL could annul its benefits and cause adverse effects on cells.

## Abbreviations

ABTS^•+^: 2,2′-azinobis-3-ethyl-benzothiazoline-6-sulfonic acid radical
AChE: acetylcholinesterase
ASChI: acetylthiocholine iodide
CBMN: cytokinesis block micronucleus assay
CBPI: cytokinesis block proliferation index
DMNQ: dimethoxy naphthoquinone
DPPH^•^: 2,2-diphenyl-1-picryl-hydrazyl radical
DTNB: 5,5′-dithio-bis (2-nitrobenzoic acid)
FAD: flavine adenine dinucleotide
FBS: foetal bovine serum
FC: Folin-Ciocalteu reagent
GR: glutathione reductase
GSH: glutathione
GSSG: glutathione disulphide
HepG2: human liver cancer cell line
HUMN: the HUman MicroNucleus project
HRP: horse radish peroxidase
HUVEC: human umbilical vein endothelial cells
IL-1β: interleukin 1β
IL-6: interleukin 6
MN: micronuclei
NADPH: nicotinamide adenine dinucleotide phosphate
NRF2: nuclear factor-erythroid factor 2-related factor 2
PAB: prooxidant-antioxidant balance
PBMC: peripheral blood mononuclear cells
PHA: phytohaemagglutinin
ROS: reactive oxygen species
SDS: sodium dodecyl sulphate
TB: trypan blue
TFC: total flavonoid content
TMB: 3,3′,5,5′-tetramethylbenzidine
TNB: 5-thio-2-nitrobenzoic acid
TNF-α: tumour necrosis factor α
TPC: total phenolic content
